# Hemodynamic Effects of Intravenous Ephedrine Administration During General Anesthesia for Radiation Therapy in Cats

**DOI:** 10.3390/vetsci13090872

**Published:** 2026-08-27

**Authors:** Daichi Seki, Seijirow Goya, Kenji Teshima, Yoshiki Yamaya

**Affiliations:** 1 Veterinary Anesthesiology & Respiratory Research Laboratory, Department of Veterinary Medicine, College of Bioresource Sciences, Nihon University, Fujisawa 252-0880, Kanagawa, Japan; brda24506@g.nihon-u.ac.jp (D.S.); teshima.kenji@nihon-u.ac.jp (K.T.); yamaya.yoshiki@nihon-u.ac.jp (Y.Y.); 2 Laboratory of Veterinary Radiology, Department of Veterinary Medicine, College of Bioresource Sciences, Nihon University, Fujisawa 252-0880, Kanagawa, Japan

**Keywords:** anesthesia, ephedrine, feline, hemodynamics, hypotension

## Abstract

Ephedrine is used as a vasopressor in cats, but the hemodynamic effects of intravenous (IV) administration have not been characterized. This study evaluated the hemodynamic effects of IV ephedrine in 10 cats that developed hypotension during anesthesia. Cats received either saline (SE) or atropine (AE) before ephedrine for hypotension (mean arterial pressure < 60 mmHg). Hemodynamic variables were analyzed using a linear mixed-effects model. No significant differences were observed between groups in the response rate (defined as mean arterial pressure ≥ 65 mmHg after ephedrine administration; SE, 70%; AE, 80%) or the duration of the pressor response (SE, 4 [1–10] min; AE, 3 [1–5] min). A significant main effect of ephedrine administration was identified for the systemic vascular resistance index, with increases observed in both groups, reaching statistical significance in the AE group. Significant interaction effects were observed for heart rate and cardiac index, both of which increased significantly in the AE group. These findings indicate that the pressor effect of IV ephedrine in anesthetized hypotensive cats is mediated primarily by increasing systemic vascular resistance index. Ephedrine provides a rapid and reliable pressor response and may be considered an early treatment option for anesthesia-induced hypotension in cats.

## Simple Summary

Ephedrine is widely used to treat hypotension in cats during general anesthesia, but its hemodynamic effects have not been well understood. This study investigated the hemodynamic effects of intravenous ephedrine in cats that developed hypotension during general anesthesia. A single intravenous bolus of ephedrine improved hypotension in 70–80% of cats. The results also showed that ephedrine increased blood pressure mainly by increasing systemic vascular resistance index. These findings improve our understanding of the cardiovascular effects of ephedrine and may help veterinarians use this drug more effectively and safely for the treatment of hypotension during general anesthesia.

## 1. Introduction

Systemic hypotension represents a clinically important cardiovascular disorder and can contribute to kidney and cardiovascular injury [1]. Systemic hypotension has been associated with acute heart failure, occurring in 16% of hospitalized dogs and cats [2]. Similarly, hypotension in hospitalized cats has been associated with increased mortality [3]. The most important point is that hypotension is a common complication in anesthetized cats [4]. In humans, intraoperative hypotension has been associated with postoperative myocardial dysfunction, delirium, stroke, and renal injury, highlighting the need for immediate and aggressive treatment [5].Therefore, further investigation of therapeutic interventions for hypotension during feline anesthesia is warranted. Recommended vasopressor and inotropic agents for maintaining adequate blood pressure in hypotensive anesthetized cats include dopamine, dobutamine, epinephrine, norepinephrine, phenylephrine, and ephedrine [6,7]. With the exception of ephedrine, these drugs are typically administered as constant-rate infusions, and several studies have already evaluated their hemodynamic effects as pressor agents for hypotension in anesthetized cats and cats with hypertrophic cardiomyopathy [8,9,10]. Ephedrine is a noncatecholamine sympathomimetic agent that exerts both direct effects on α- and β-adrenergic receptors and indirect effects through the release of endogenous norepinephrine [11,12]. Intramuscular and intravenous (IV) bolus administration of ephedrine is widely recognized as a standard treatment for hypotension during general anesthesia in cats [6,7,13,14]. However, emergency IV bolus administration may be preferable, as prophylactic intramuscular administration of ephedrine delays but does not prevent the onset of hypotension after anesthetic induction in cats [12,14]. Despite its clinical use, information regarding IV bolus administration of ephedrine in cats remains limited, and detailed hemodynamic data, including cardiac output (CO) and systemic vascular resistance, are lacking. Furthermore, a previous retrospective study in anesthetized cats reported that atropine premedication did not influence the pressor response to ephedrine [14]. Therefore, further evaluation of the hemodynamic effects and autonomic responses to IV bolus ephedrine is required in anesthetized cats.

The present study investigated the effects of IV bolus ephedrine administration on hemodynamics and autonomic balance in anesthetized cats using echocardiography and a parasympathetic tone activity (PTA) monitor, which may be useful for assessing sympathetic activation and relative reductions in parasympathetic tone within the autonomic nervous system [15]. We hypothesized that IV bolus administration of ephedrine increases blood pressure in anesthetized cats through positive inotropic and vasoconstrictive effects in addition to its positive chronotropic effects mediated by the autonomic nervous system.

## 2. Materials and Methods

### 2.1. Animals

This was a single-center, prospective, randomized crossover, clinical study that included client-owned cats referred to Nihon University Animal Medical Center between April 2025 and April 2026 for radiation therapy (RT; Versa HD linear accelerator system, Elekta K.K., Tokyo, Japan). All cats were scheduled to receive four or six fractions of RT, with a minimum interval of 1 week between treatment sessions. Accordingly, the study protocol was performed during two scheduled anesthetic sessions for RT, with a washout period of at least 1 week between treatments. Before anesthesia, all cats underwent comprehensive pre-anesthetic evaluation, including physical examination, complete blood count, serum biochemical analysis, electrocardiogram (ECG; Cardisuny D800, FUKUDA M E KOGYO Co., Ltd., Tokyo, Japan), noninvasive blood pressure measurement (BP100D II, FUKUDA M E KOGYO Co., Ltd., Tokyo, Japan), and echocardiography (Vivid E95, GE Healthcare Japan Corp., Tokyo, Japan). Cats classified as American Society of Anesthesiologists Physical Status (ASA-PS) II based on these evaluations were eligible for enrollment. Cats diagnosed with cardiac disease, arrhythmias, or hypertension during the pre-anesthetic screening were excluded. In addition, cats with brain tumors or intracranial invasion of head tumors identified via computed tomography (Aquilion ONE, Canon Medical Systems, Tochigi, Japan) or magnetic resonance imaging (Vantage Centurian, Canon Medical Systems, Tochigi, Japan) were excluded. This study was conducted as a clinical trial involving client-owned animals, with the approval of the Nihon University Animal Medical Center Research and Trial Ethics Committee (approval number: ANMEC-1-025; approval date: 4 April 2025). Institutional consent forms included provisions permitting the use of clinical data for research purposes, and informed consent was obtained from all owners prior to study enrollment. No AI-assisted technologies were used in the generation of this manuscript.

### 2.2. Anesthesia and Instrumentation

All cats were fasted for at least 12 h, and water was withheld for 2 h before anesthesia for RT. Following the pre-anesthetic examination, an appropriately sized catheter (Surflo F&F, TERUMO, Tokyo, Japan) was placed in the right or left cephalic vein. In cats showing clinical signs of dehydration during the pre-anesthetic screening, IV fluid therapy with lactated Ringer’s solution (lactated Ringer’s solution “FUSO,” Fuso Pharma. Ind. Ltd., Osaka, Japan) was administered for an appropriate period before anesthetic induction. The cats were then transferred to the anesthesia induction room and allowed to acclimatize for 10–15 min before induction. Prior to anesthetic induction, cats received IV administration of saline (OTSUKA NORMAL SALINE, Otsuka Pharma. Co., Ltd., Tokyo, Japan) or atropine (ATROPINE SULFATE Injection 0.5 mg, NIPRO ES PHARMA Co., Ltd., Osaka, Japan). Saline was administered at a volume of 0.04 mL/kg to match the injection volume of atropine. Atropine was administered at a dose of 0.02 mg/kg. Anesthesia was induced 5 min after saline or atropine administration to allow sufficient time for drug effects to develop. Anesthesia was induced with propofol (PropoFlo28, Zoetis Japan, Tokyo, Japan), initially administered as an IV bolus of 1.5 mg/kg, followed by slow IV administration to effect. Once reduced jaw tone was confirmed, topical anesthesia was applied bilaterally to the arytenoid cartilages to prevent laryngospasms. Specifically, one drop of lidocaine (Xylocaine 2% for Intravenous Injection, Sandoz Pharma K.K., Tokyo, Japan) was applied to each arytenoid cartilage using a 24-gauge catheter sheath (Surflo F&F, TERUMO, Tokyo, Japan) attached to a 1 mL syringe (TERUMO Syringe 1 mL, TERUMO, Tokyo, Japan) [16,17]. One minute after topical anesthesia, tracheal intubation was performed using a laryngoscope and an appropriately sized cuffed endotracheal tube (SHERIDAN Endotracheal Tube, Teleflex Medical Japan, Tokyo, Japan) once the palpebral reflex and jaw tone had disappeared. General anesthesia was maintained using a semi-closed-circuit anesthesia machine (NS-3000, Acoma Medical Industry Co., Ltd., Tokyo, Japan) with isoflurane (DS isoflurane, Bussan Animal Health Co., Ltd., Osaka, Japan) in 100% oxygen at a flow rate of 0.8–2.0 L/min. The isoflurane concentration was adjusted to the minimum level required to abolish the palpebral reflex and jaw tone. Mechanical ventilation was provided throughout anesthesia. In accordance with the manufacturer’s recommendation of a respiratory rate of at least 8 breaths/min, the ventilator was set to pressure-controlled ventilation with an inspiratory pressure of 8 cmH_2_O and a respiratory rate of 8 breaths/min [15,18]. Body temperature was maintained using standard blankets. Physiological variables, including heart rate (HR), percutaneous arterial oxygen saturation (SpO_2_), partial pressure of end-tidal carbon dioxide (PETCO_2_), respiratory rate, and inspiratory and expiratory isoflurane concentrations (FIiso and FEiso, respectively), were continuously monitored (AM140; FUKUDA M E KOGYO Co., Ltd., Tokyo, Japan) and recorded. Noninvasive blood pressure, such as systolic arterial pressure (SAP), mean arterial pressure (MAP), and diastolic arterial pressure (DAP), was measured using an oscillometric device (AM140, FUKUDA M E KOGYO Co., Ltd., Tokyo, Japan). An appropriately sized cuff, with a width approximately 40% of the circumference of the measurement site, was placed either at the base of the tail or over the dorsal pedal artery [17]. The accuracy of this oscillometric device has been routinely validated in our clinical setting by comparisons with invasive arterial blood pressure measurements, demonstrating consistent agreement [19]. In addition, oscillometric blood pressure monitors employing algorithms similar to that used in the device in the present study have been used for blood pressure measurement in healthy cats and are also used in clinical practice [20]. Electrode clips from the PTA monitor (HEIWA BUSSAN CO., Ltd., Tokyo, Japan) were attached to the skin of the right and left forelimbs and left hindlimbs. The attachment sites were moistened with isopropyl alcohol to improve ECG detection sensitivity. The principles and functions of the PTA monitor have been described previously [21]. Briefly, the PTA monitor used a lead II ECG obtained from electrode clips attached to the animal. HR variability frequency analysis was performed using ECG-derived R–R, with emphasis on high-frequency components associated with parasympathetic activity. The PTA index was calculated using a unique algorithm based on the area under the R–R interval series corresponding to the parasympathetic tone-series curve. PTA indices range from 0 to 100, with higher values indicating greater parasympathetic activity, whereas lower values indicate reduced parasympathetic activity and/or increased sympathetic activity. In the present study, the PTAi, calculated from the preceding 54 PTA index values and updated every second, was recorded and used for statistical analysis [22,23]. Echocardiography was performed using a 5-MHz sector probe connected to an ultrasound diagnostic system (Vivid E95, GE Healthcare Japan Corp., Tokyo, Japan).

### 2.3. Study Design

Cats were assigned to either the saline–ephedrine group (SE group) or the atropine–ephedrine group (AE group). In this crossover study, cats were randomly allocated to receive either saline or atropine in the first study session using a lottery method. During the second study session, each cat received the alternate treatment. The study procedures were conducted by two investigators: an operator and a recorder. The operator prepared the lottery slips, and a third party who was not involved in or aware of the study procedures drew a slip to determine whether saline or atropine was administered during the first study session. The allocation was known only to the operator, and the recorder remained blinded to the allocation. Cats that developed hypotension, defined as MAP < 60 mmHg for 5 min [17,24], after stabilization of anesthetic depth and completion of anesthetic monitoring, PTA monitoring, and echocardiographic preparation, were included in the study protocol. As an initial intervention for hypotension, isoflurane was gradually reduced to the minimum anesthetic depth at which the palpebral reflex and jaw tone disappeared. This anesthetic depth was required for all cats to allow transfer from the anesthesia induction room to the RT room. Then, lactated Ringer’s solution (2 mL/kg) was administered slowly for 1 min. The study proceeded only in cats that remained hypotensive after fluid administration. The recorder obtained baseline data (BL), including anesthetic variables (SpO_2_, PETCO_2_, respiratory rate, FIiso, and FEiso); hemodynamic variables (HR, SAP, MAP, and DAP); stroke volume (SV) and CO from echocardiography; and PTAi from the PTA monitor. SV and CO were calculated automatically using echocardiographic measurements of the aortic annulus diameter in the left apical five-chamber view and the velocity–time integral by tracing the left ventricular outflow tract ejection velocity waveform using pulse-wave Doppler echocardiography. All measurements were performed by the same operator, and the average value of three consecutive heartbeats was used for analysis. After BL measurements had been recorded, ephedrine (EPHEDRINE “NAGAI” Injection, Nichi-Iko Pharma. Co., Ltd., Toyama, Japan) was administered as an IV bolus of 0.05 mg/kg. Ephedrine was prepared by diluting 1 mL of the stock solution with 39 mL of saline. After ephedrine administration, the anesthetic variables, hemodynamic variables, SV, CO, and PTAi were recorded at 1-min intervals. Blood pressure measurements (SAP, MAP, and DAP) were obtained continuously, and the values from the measurement cycle closest to each recording time point were used for analysis. Recordings were performed for at least 5 min and continued for up to 10 min or until MAP decreased to <65 mmHg. The study protocol was terminated when recording was completed. Subsequently, anesthesia was maintained according to the standard institutional protocol, and RT was administered.

### 2.4. Data Collection

Anesthetic variables, hemodynamic variables, SV, CO, and PTAi were extracted from the anesthetic records and used for statistical analysis. The stroke volume index (SVI) and cardiac index (CI), calculated by normalizing SV and CO to body surface area (calculated as 0.1 × body weight ^2/3^), were subsequently used for analysis. The systemic vascular resistance index (SVRI) was calculated using the simplified formula (MAP/CI) × 79.9 and was used for analysis [25,26]. Data regarding signalment, underlying diseases, clinical status, and complications associated with the study protocol were collected from the electronic medical record system.

### 2.5. Statistical Analysis

The required sample size was calculated using EZR (Saitama Medical Center, Jichi Medical University), a graphical user interface for R, version 4.4.0 (The R Foundation for Statistical Computing). All other statistical analyses were conducted using commercially available software (Prism, version 9.0; GraphPad Software Inc., Boston, MA, USA). Normality was assessed using the Shapiro–Wilk test. The number of radiation therapy sessions was compared between the SE and AE groups using the Wilcoxon signed-rank test. Anesthetic variables, including SpO_2_, PETCO_2_, FIiso, FEiso, propofol dose, and interval between atropine and ephedrine administration, were compared between the SE and AE groups. Comparisons between two groups were performed using either the paired *T*-test or Wilcoxon signed-rank test, as appropriate. Based on the existing guidelines [7], MAP < 60 mmHg was interpreted as hypotension requiring treatment, 60–64 mmHg as the range in which treatment should be considered, and 65–70 mmHg as the range appropriate for continued monitoring. Therefore, because MAP ≥ 65 mmHg is above the range in which treatment should be considered, the response rate to ephedrine was defined as the achievement of MAP ≥ 65 mmHg after ephedrine administration. Response rates were calculated and compared between the SE and AE groups using Fisher’s exact test. Among responders, the duration for which MAP remained ≥65 mmHg from the BL was compared between groups using the Mann–Whitney U test. Hemodynamic variables (HR, SAP, MAP, DAP, SVI, CI, and SVRI) and PTAi from BL to 5 min following ephedrine administration were analyzed using a linear mixed-effects model. In this model, ephedrine administration, atropine premedication, and their interactions were included as fixed effects, whereas individual cats were treated as random effects. Tukey’s multiple comparison was performed to determine the presence of significant main effects or interactions. For detailed hemodynamic analysis, the relative change from BL were calculated for variables contributing to blood pressure, including HR, SVI, CI, and SVRI. Relative changes were analyzed using the aforementioned linear mixed-effects model. Based on previous reports, the baroreceptor reflex was defined as a decrease in HR of ≥20% from BL following ephedrine administration [14]. Statistical significance was defined as *p* < 0.05. Data are presented as mean ± standard deviation or median (range), as appropriate.

## 3. Results

Based on previous reports, a sample size of 10 cats was calculated to detect a mean difference in MAP of 15 mmHg between BL (before ephedrine administration) and each time point after ephedrine administration, assuming a standard deviation of 15 mmHg, a two-sided significance level of 0.05, and a statistical power of 0.80 [15]. Accordingly, 10 cats were enrolled in the study. The study population included four cats with unilateral intranasal adenocarcinoma, four with unilateral intranasal lymphoma, one with left maxillary melanoma, and one with left mandibular squamous cell carcinoma. The breeds included mixed-breed cats (*n* = 9) and one Norwegian forest cat (*n* = 1). The median age was 14 (6–17) years, the mean weight was 3.8 ± 0.9 kg, and the median body condition score was 3 (2–4)/5. Four cats were neutered males and six were spayed females. All cats were classified as ASA-PS II based on pre-anesthetic evaluation, with no evidence of cardiac disease, arrhythmia, or hypertension. Although nasopharyngeal invasion and bone destruction were observed on pre-RT computed tomography, intracranial invasion was not detected in any of the cats. One cat had a history of hyperthyroidism, which was well controlled with thiamazole; pre-anesthetic free thyroxine, thyroxine, and thyroid-stimulating hormone levels were within the respective reference ranges, and there were no associated clinical signs. The two cats received intravenous administration of lactated Ringer’s solution at a rate of 3 mL/kg/h for 1 h before all general anesthesia procedures. Therefore, no significant differences were observed between the two groups in the number of cats receiving fluid therapy or the total volume of fluid administered. The study protocol was performed at a median radiation therapy session number of 2.5 [1–4] in the SE group and 2.5 [1–4] sessions in the AE group, with no significant difference between the groups (median of differences = 1.000; 95% confidence interval = −1.000 to 1.000; *p* > 0.999). The study protocol was first performed in the SE group in six cats and in the AE group in four vats. The interval between study protocol sessions was 7 [7–14] days, and the interval between all general anesthesia sessions, regardless of whether the study protocol was performed, was also 7 [7–14] days. Although the treatment effect of radiation therapy during each session was not evaluated, based on the above findings, the effects of differences in session number and treatment intervals between the groups were unlikely to have substantially influenced the results. Oscillometric blood pressure measurements were obtained from the tail artery in 9 cats and from the dorsal pedal artery in 1 cat. The measurement site was kept consistent between the SE and AE groups for each cat.

Anesthetic variables showed no significant differences between the SE and AE groups, including propofol dose (8.0 [7.0–10] mg/kg vs. 7.5 [5.0–10] mg/kg; median of differences = 0.5000; 95% confidence interval = 0.000 to 2.000; *p* = 0.125), FIiso (2.0 [1.8–2.4]% vs. 2.1 [1.9–2.2]%; median of differences = −0.1000; 95% confidence interval = −0.2000 to 0.2000; *p* = 0.4063), FEiso (1.7 [1.6–1.7]% vs. 1.7 [1.6–1.9]%; median of differences = 0.000; 95% confidence interval = −0.1000 to 0.000; *p* = 0.2500), SpO_2_ (100 [99–100]% vs. 100 [98–100]%; median of differences = 0.000; 95% confidence interval = 0.000 to 1.000; *p* = 0.0850), PETCO_2_ (26 [21–29] mmHg vs. 25 [23–38] mmHg; median of differences = −0.5000; 95% confidence interval = −2.000 to 2.000; *p* = 0.9570), and the interval between atropine and ephedrine administration (14 [9–21] vs. 15 [13–23] min; median of differences = −2.000; 95% confidence interval = −6.000 to 2.000; *p* = 0.1719). All cats developed hypotension following anesthesia induction. Isoflurane reduction and administration of a 2 mL/kg bolus of lactated Ringer’s solution were performed in all cats; however, hypotension did not improve after these interventions. At BL, before ephedrine administration, only HR was significantly higher in the AE group than in the SE group (mean of differences = −23.30; 95% confidence interval = −40.56 to 6.044; *p* = 0.0014), whereas no significant differences were observed in the other variables (Table 1).

The response rates to ephedrine were 70% (7/10) in the SE group and 80% (8/10) in the AE group, with no significant difference between the two groups (odds ratio = 0.5833; 95% confidence interval = 0.08665 to 3.649; *p* > 0.9999). The duration for which MAP remained ≥ 65 mmHg following ephedrine administration was 4 min (1–10 min) in the SE group and 3 min (1–5 min) in the AE group, with no significant difference (median of differences = 1.000; 95% confidence interval = −2.000 to 3.000; *p* = 0.880). Regarding blood pressure variables, SAP, MAP, and DAP demonstrated a significant main effect of ephedrine administration, whereas no significant interaction with atropine premedication was observed. MAP increased significantly at 1 min (mean of differences = −17.00; 95% confidence interval = −32.12 to 1.883; *p* = 0.014) and 2 min (mean of differences = −17.60; 95% confidence interval = −32.72 to 2.483; *p* = 0.009) after ephedrine administration in the SE group and at 1 min (mean of differences = −26.40; 95% confidence interval = −41.52 to −11.28; *p* ≤ 0.001), 2 min (mean of differences = −26.90; 95% confidence interval = −42.02 to −11.78; *p* ≤ 0.001), 3 min (mean of differences = −23.90; 95% confidence interval = −39.02 to −8.783; *p* ≤ 0.001), and 4 min (mean of differences = −16.10; 95% confidence interval = −31.22 to −0.9834; *p* ≤ 0.027), in the AE group. No significant differences in MAP were observed between groups at corresponding time points following ephedrine administration (Table 1 and Table 2).

**Figure 1:**
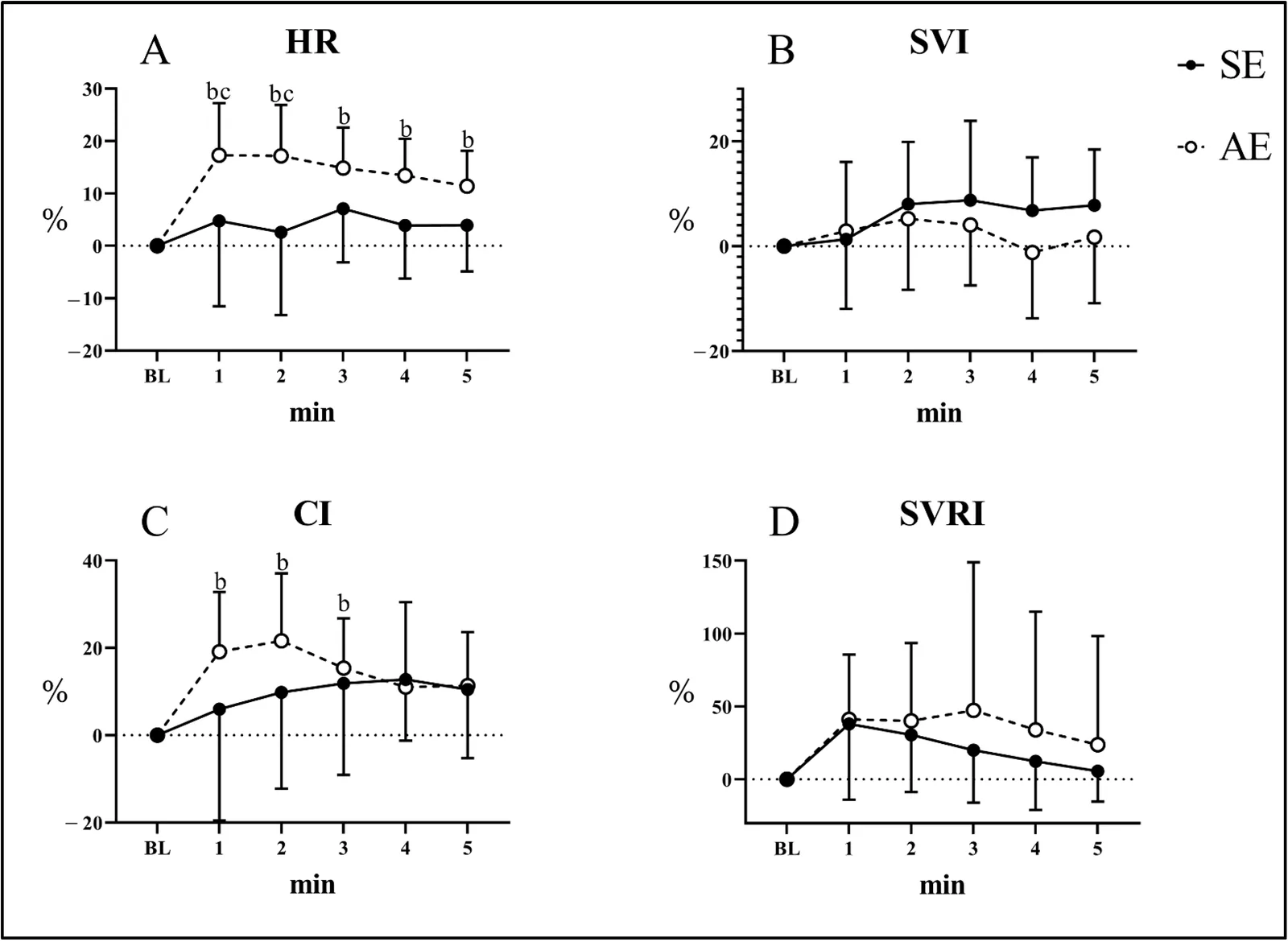
(**A**–**D**) Relative changes in HR, SVI, CI, and SVRI following ephedrine administration. Abbreviations: AE, atropine–ephedrine group; BL, baseline; CI, cardiac index; HR, heart rate; SE, saline–ephedrine group; SVI, stroke volume index; SVRI, systemic vascular resistance index. Hemodynamic effects of intravenous ephedrine (0.05 mg/kg) in 10 anesthetized cats. Saline (0.04 mL/kg) was administered intravenously before ephedrine in the SE group, whereas atropine (0.02 mg/kg) was administered intravenously before ephedrine in the AE group. Line graphs show mean values with markers representing data points and error bars indicating standard deviations. ^b^ Indicates a significant difference (*p* < 0.05) compared with BL in the AE group. ^c^ Indicates a significant difference (*p* < 0.05) between groups at the corresponding time point. The solid and dashed lines represent the SE and AE groups, respectively.

Significant main effects of ephedrine and its interaction with atropine were observed for HR. No significant change was observed in the SE group, whereas the AE group showed a sustained significant increase in HR after ephedrine administration. HR values were significantly higher in the AE group than in the SE group at corresponding post-administration time points. Similar findings were observed for the relative changes, with a significant maximum increase of 17 ± 9% (1 min; mean of differences = −17.33; 95% confidence interval = −26.61 to −8.045; *p* ≤ 0.001) in the AE group, whereas no significant increase was observed in the SE group. No significant main effect of ephedrine or its interaction with atropine was observed for SVI, and no significant changes were identified after ephedrine administration. Similar findings were observed for the relative changes, although increases tended to occur between 2 and 3 min after ephedrine administration rather than at 1 min. Significant main effects of ephedrine and its interaction with atropine were observed for CI. No significant change in CI was observed in the SE group, whereas significant increases were observed at 1 min (mean of differences = −0.3600; 95% confidence interval = −0.6384 to −0.08162; *p* = 0.002), 2 min (mean of differences = −0.3770; 95% confidence interval = −0.6554 to −0.09862; *p* = 0.001), and 3 min (mean of differences = −0.2980; 95% confidence interval = −0.5764 to −0.01962; *p* = 0.025) in the AE group. No significant difference in the CI was observed between groups at the corresponding time points after ephedrine administration. Similar results were observed for the relative changes, with a significant increase at 1 min (19 ± 14%; mean of differences = −19.13; 95% confidence interval = −34.34 to −3.926; *p* = 0.003), 2 min (22 ± 15%; mean of differences = −21.65; 95% confidence interval = −36.86 to −6.441; *p* ≤ 0.001), 3 min (15 ± 11%; mean of differences = −15.37; 95% confidence interval = −30.58 to −0.1655; *p* = 0.045) in the AE group. In the SE group, CI gradually increased over time and reached a maximum increase of 13 ± 17% at 4 min (mean of differences = −12.75; 95% confidence interval = −27.96 to 2.457; *p* = 0.192); however, this change was not statistically significant. A significant main effect of ephedrine administration was observed for SVRI, with a significant increase identified only in the AE group 1 min (mean of differences = −918.7; 95% confidence interval = −1810 to −27.48; *p* = 0.037) after ephedrine administration. Similar findings were observed for the relative changes, and no significant between-group difference was observed; however, increases of 38 ± 49% (mean of differences = −38.03; 95% confidence interval = −86.41 to 10.35; *p* = 0.275) in the SE group and 41 ± 42% (mean of differences = −41.23; 95% confidence interval = −89.61 to 7.153; *p* = 0.174) in the AE group were observed at 1 min after ephedrine administration. Significant main effects of both ephedrine administration and atropine premedication were observed for PTAi, whereas no significant interaction was identified. Significant decreases in PTAi were observed in both groups 1 min after ephedrine administration, with no significant between-group difference (mean of differences = 11.80; 95% confidence interval = −12.02 to 35.62; *p* = 0.864) (Figure 1, Table 1, Table 2 and Table 3).

No arrhythmias were observed during the study period. All cats underwent RT according to the study protocol and recovered from anesthesia without complications. No anesthetic complications were detected. Additionally, a baroreceptor reflex was observed in two cats in the SE group. HR decreased from 114 to 86 beats/min and 124 to 97 beats/min following ephedrine administration, whereas adequate pressor responses were maintained (MAP: 52 to 70 mmHg and 34 to 64 mmHg, respectively).

## 4. Discussion

The present study suggests that IV bolus administration of ephedrine provides an effective pressor response in anesthetized cats, regardless of atropine. Consistent with a response rate of 55–69% during anesthetized examinations or RT in a previous study [14], the response rate observed in the present study was higher at approximately 70–80%, indicating that a substantial proportion of cats achieved the target blood pressure. All cats developed hypotension immediately after anesthetic induction and required therapeutic intervention. However, hypotension did not improve after isoflurane reduction and intravenous bolus administration of lactated Ringer’s solution, despite both interventions being commonly recommended as an initial treatment for hypotension in anesthetized cats [7,11]. Because the present study involved clinical cases, the efficacy of ephedrine could not be evaluated against a control group that received no pharmacological intervention. However, the findings of the present study suggested that IV bolus ephedrine may serve as a useful second-line emergency treatment for hypotension immediately after anesthetic induction in cats prior to initiating continuous rate infusions, because of its relatively consistent pressor responses.

The pressor effect of ephedrine in anesthetized cats appears to be mediated predominantly by vasoconstriction. To the authors’ knowledge, this is the first study to evaluate hemodynamic changes following IV bolus ephedrine administration in anesthetized cats using echocardiography. Among the evaluated variables, only SVRI showed consistently high relative changes in both groups. In the present study, ephedrine exerted a significant main effect on SVRI, with a significant increase observed in the AE group. Although ephedrine administration had a significant main effect, the absence of a significant difference in the SE group was likely attributable to the large variability, as reflected by the large standard deviations. Notably, some cats that failed to achieve MAP ≥ 65 mmHg demonstrated low relative changes in SVRI following ephedrine administration (two of three cats in the SE group: −0.3% and 7.0%; two cats in the AE group: 0.09% and 19%). These findings may suggest the importance of the vasoconstrictive effect of ephedrine. In contrast, no significant changes in SVI or CI were observed in the SE group. The SVI and CI reflect left ventricular systolic performance but do not directly assess left ventricular systolic function or contractility [27]. Because left ventricular systolic performance is influenced by afterload, the vasoconstrictive effects of ephedrine may have masked potential increases in SVI and CI. This observation should be interpreted cautiously, but it appears consistent with the possibility that the early increase in SVRI limited the increase in SVI after ephedrine administration. Consequently, although overt improvements in SVI and CI were not observed, a positive inotropic effect of ephedrine cannot be excluded. In the AE group, although SVI showed no significant changes, CI demonstrated significant main effects of ephedrine and significant interaction effects with atropine. These findings suggest that atropine premedication augmented the positive chronotropic effects of ephedrine. Similarly, no significant changes in HR were observed in the SE group, whereas significant increases in HR were observed in the AE group following ephedrine administration. The AE group exhibited higher blood pressure than that in the SE group; HR and CI were significantly higher in the AE group than in the SE group. However, compared to previous reports in anesthetized dogs [15], increases in both CI values and relative changes appeared shorter in duration. Moreover, no significant differences were observed in the response rate or the time maintained at MAP ≥ 65 mmHg, indicating that the impact of atropine premedication for pressor response is limited in anesthetized cats.

Baroreceptors respond to increased arterial blood pressure by transmitting impulses via the glossopharyngeal and vagus nerves to the nucleus tractus solitarius within the central nervous system. These signals initiate compensatory autonomic responses characterized by enhanced parasympathetic activity and suppression of peripheral sympathetic activity, thereby restoring blood pressure to normal levels [28,29]. A previous study in anesthetized dogs reported that ephedrine attenuates its own pressor effect by inducing a reduction in HR through baroreceptor reflex-mediated vagal activation [15]. In the present study, decreases in HR were generally not observed following ephedrine administration, and PTAi demonstrated no significant interaction with atropine premedication. Collectively, these findings suggest that the pressor effect of ephedrine in anesthetized cats is minimally affected by parasympathetic tone, which likely explains the limited impact of atropine premedication. These findings suggest that species-specific hemodynamic management is required in anesthetized dogs and cats.

The present study had some limitations. First, the cats included in our study were not healthy experimental animals but rather had neoplastic diseases requiring RT. Therefore, the present findings may not be directly generalizable to healthy young cats or cats with different underlying diseases. The present study included cats with neoplastic diseases that exhibited decreased appetite and a tendency toward weight loss compared with healthy cats, as well as some degree of dehydration, although dehydration was corrected as much as possible by intravenous fluid administration. Given that dehydration and low body weight may be risk factors for hypotension, the inclusion of cats with neoplastic diseases may have influenced the hemodynamic responses to ephedrine [30]. Advanced age has been identified as a potential risk factor for anesthesia-associated hypotension, and the severity of hypotension observed in the present study appeared greater than that reported previously for IV ephedrine administration in cats [14,31]. The use of propofol alone at a relatively high dose may also have contributed to the development of hypotension after anesthetic induction. Although it remains unclear whether reducing the propofol requirement decreases the incidence of hypotension, previous reports have suggested that reducing the propofol requirement through the use of preanesthetic medication could be advantageous in cats [32]. Although ephedrine demonstrated an effective pressor response in the present study, the severe hypotension observed in our study may also have made achievement of MAP ≥ 65 mmHg more difficult, potentially resulting in underestimation of the true pressor response rate for ephedrine. Furthermore, only a single ephedrine dose (0.05 mg/kg) was evaluated in this study, and higher doses may have produced more effective pressor responses. However, because initial administration of a high dose is associated with the risk of excessive increases in blood pressure, initiating ephedrine at 0.025–0.05 mg/kg and gradually titrating the dose is recommended [14]. Second, the effects of various atropine doses and administration routes were not evaluated. Although 0.02 mg/kg IV atropine effectively increased HR in all cats, higher doses may have augmented the pressor effects of ephedrine further. The recommended atropine dose in cats and dogs is 0.02–0.04 mg/kg, administered either IV or intramuscularly [33]. Therefore, although the atropine protocol used in the present study was clinically appropriate, the interaction between atropine and ephedrine may vary according to dose and route of administration. Third, blood pressure was measured using an oscillometric method rather than invasive arterial monitoring. In the present study, all cats developed hypotension immediately after anesthesia induction, necessitating timely intervention. Given the need for anesthetic stabilization and equipment preparation, placement of an arterial catheter for invasive monitoring was considered challenging. In feline blood pressure monitoring, the oscillometric method is generally regarded as reliable when cuff size and attachment site are appropriate [17]. The accuracy of oscillometric blood pressure measurement compared with invasive arterial blood pressure measurement in cats has recently been evaluated in a systematic review. Among the 11 studies evaluating oscillometric methods, two found them to be comparable with invasive measurements, five found them to be comparable under specific conditions, whereas four did not find them to be comparable. Ultimately, the review concluded that, under appropriate conditions, oscillometric blood pressure measurement may provide results comparable to invasive arterial blood pressure measurement, particularly for MAP [34]. These findings support the clinical utility of oscillometric blood pressure measurement in anesthetized cats. However, they do not completely exclude the possibility of measurement error associated with the oscillometric method. Therefore, the blood pressure measurements obtained in the present study may have differed from those that would have been obtained using invasive arterial blood pressure measurement. Arterial catheterization is generally considered the reference standard for blood pressure monitoring; however, this technique is technically demanding and is infrequently performed in routine clinical feline cases [17]. Therefore, invasive blood pressure monitoring may be less practical for routine, short-term anesthesia, such as RT. Given the clinical importance of oscillometric blood pressure assessment, continued efforts to improve its reliability through comparison with invasive arterial measurements remain clinically important. Fourth, administration of anticholinergic agents may have influenced PTAi measurements. According to the manufacturer’s instructions, anticholinergic agents and arrhythmias may affect PTAi assessment during nociceptive monitoring with the PTA monitor. However, the present study did not aim to evaluate nociceptive stimuli, and previous research reported no significant effect of atropine administration on PTAi measurements [15]. Reports regarding PTA monitoring in cats remain limited, and previous studies have described challenges including inconsistent signal acquisition and limited reference data [35]. Additionally, ECG leads other than lead II were used in some cats to ensure clear visualization of the R–R intervals. Nevertheless, PTAi changes in these cats showed patterns similar to those observed in the remaining study population following ephedrine administration, suggesting that PTA monitoring retained acceptable interpretability. Further studies are warranted to clarify the clinical utility and characteristics of PTA monitoring in cats. Fifth, the SVI and CI were measured using non-invasive echocardiography in the present study. Catheter-based techniques are generally considered the gold standard for measuring SVI and CI, and a previous study reported incomplete agreement between echocardiographic and catheter-derived measurements. Therefore, although catheterization-based measurements and echocardiography show a strong linear relationship, accurate assessment of changes in SVI and CI requires consideration of catheter-based measurements [36]. Finally, although the sample size was determined based on an a priori sample size calculation, the inclusion of only 10 cats represents a limitation of this study. The relatively small sample size may have limited the statistical power to detect differences in hemodynamic variables, thereby increasing the risk of Type II error. However, a linear mixed-effects model was used to account for interindividual variability among cats in the statistical analysis.

## 5. Conclusions

The present study demonstrated that ephedrine exerts a pressor effect mainly by increasing SVRI in cats experiencing anesthesia-induced hypotension. Atropine premedication for the pressor response was minimal, because the HR and CI have a limited impact on the overall pressor response in hypotensive cats being treated with ephedrine.

## Figures and Tables

**Table 1:** Changes in hemodynamic variables and PTAi following ephedrine administration.

Variables	Group	BL	1 min	2 min	3 min	4 min	5 min
HR	SE	123 ± 18	128 ± 22	125 ± 22	130 ± 13	126 ± 15	126 ± 13
(beats/min)	AE	146 ± 14 ^c^	171 ± 14 ^b c^	170 ± 14 ^b c^	167 ± 13 ^b c^	165 ± 13 ^b c^	162 ± 13 ^b c^
SAP	SE	73 ± 7.7	91 ± 11 ^a^	93 ± 19 ^a^	89 ± 24	86 ± 25	81 ± 22
(mmHg)	AE	72 ± 9.2	99 ± 12 ^b^	100 ± 11 ^b^	96 ± 25 ^b^	90 ± 20	80 ± 16
MAP	SE	49 ± 5.7	66 ± 9.7 ^a^	66 ± 11 ^a^	63 ± 14	60 ± 14	56 ± 9.8
(mmHg)	AE	45 ± 9.0	72 ± 11 ^b^	72 ± 9.6 ^b^	69 ± 24 ^b^	61 ± 19 ^b^	56 ± 13
DAP	SE	38 ± 4.5	54 ± 9.8 ^a^	53 ± 9.4 ^a^	49 ± 11	45 ± 9.1	43 ± 7.2
(mmHg)	AE	35 ± 8.0	58 ± 12 ^b^	59 ± 11 ^b^	56 ± 23 ^b^	49 ± 18 ^b^	45 ± 12
SVI	SE	14.4 ± 5.3	14.4 ± 4.6	15.6 ± 5.8	15.7 ± 6.0	15.4 ± 6.0	15.6 ± 6.1
(ml/m^2^)	AE	13.9 ± 6.8	13.8 ± 6.2	14.0 ± 6.1	14.2 ± 6.7	13.0 ± 5.5	13.6 ± 6.2
CI	SE	1.73 ± 0.53	1.80 ± 0. 61	1.87 ± 0.57	1.91 ± 0.61	1.93 ± 0.62	1.90 ± 0.62
(L/min/m^2^)	AE	1.97 ± 0.88	2.33 ± 1.03 ^b^	2.35 ± 0.98 ^b^	2.27 ± 1.02 ^b^	2.11 ± 0.81	2.14 ± 0.90
SVRI	SE	2513 ± 1007	3226 ± 1155	3111 ± 1071	2901 ± 1155	2761 ± 1175	2602 ± 996
(dyne × s/cm^5^/m^2^)	AE	2297 ± 1247	3216 ± 2184 ^b^	3044 ± 1679	3063 ± 1749	2714 ± 1310	2567 ± 1334
PTAi	SE	67 ± 19	42 ± 19 ^a^	65 ± 13	70 ± 16	74 ± 11	74 ± 14
	AE	63 ± 15	30 ± 13 ^b^	54 ± 17	53 ± 21	58 ± 22	65 ± 16

Abbreviations: AE, atropine–ephedrine group; BL, baseline; CI, cardiac index; DAP, diastolic arterial pressure; HR, heart rate; MAP, mean arterial pressure; PTAi, immediate parasympathetic tone activity; SAP, systolic arterial pressure; SE, saline–ephedrine group, SVI, stroke volume index; SVRI, systemic vascular resistance index. Hemodynamic effects of intravenous ephedrine (0.05 mg/kg) in 10 anesthetized cats. Saline (0.04 mL/kg) was administered intravenously before ephedrine in the SE group, whereas atropine (0.02 mg/kg) was administered intravenously before ephedrine in the AE group. Data are presented as mean ± standard deviation. Significant differences represent the results of Tukey’s multiple comparisons test following a linear mixed-effects model. ^a^ (SE group) and ^b^ (AE group) indicate statistical significance (*p* < 0.05) compared with BL. ^c^ indicates statistical significance (*p* < 0.05) between two groups at the same time point.

**Table 2:** Results of a linear mixed-effects model for hemodynamic variables and PTAi.

Variables	Fixed Effects	*p* Value
HR	ephedrine administration	<0.001 ^a^
	atropine premedication	<0.001 ^a^
	interaction	<0.001 ^a^
SAP	ephedrine administration	<0.001 ^a^
	atropine premedication	0.439
	interaction	0.538
MAP	ephedrine administration	<0.001 ^a^
	atropine premedication	0.273
	interaction	0.395
DAP	ephedrine administration	<0.001 ^a^
	atropine premedication	0.084
	interaction	0.327
SVI	ephedrine administration	0.123
	atropine premedication	0.212
	interaction	0.077
CI	ephedrine administration	0.004 ^a^
	atropine premedication	0.059
	interaction	0.006 ^a^
SVRI	ephedrine administration	0.008 ^a^
	atropine premedication	0.920
	interaction	0.813
PTAi	ephedrine administration	<0.001 ^a^
	atropine premedication	0.042 ^a^
	interaction	0.571

Abbreviations: CI, cardiac index; DAP, diastolic arterial pressure; HR, heart rate; MAP, mean arterial pressure; PTAi, immediate parasympathetic tone activity; SAP, systolic arterial pressure; SVI, stroke volume index; SVRI, systemic vascular resistance index. In this model, ephedrine administration, atropine premedication, and their interaction were included as fixed effects, with individual subjects treated as a random effect. ^a^ indicates statistical significance (*p* < 0.05).

**Table 3:** Results of a linear mixed-effects model for relative changes in HR, SVI, CI, and SVRI.

Variables	Fixed Effects	*p* Value
HR	ephedrine administration	<0.001 ^a^
	atropine premedication	<0.005 ^a^
	interaction	<0.001 ^a^
SVI	ephedrine administration	0.068
	atropine premedication	0.436
	interaction	0.266
CI	ephedrine administration	0.002 ^a^
	atropine premedication	0.298
	interaction	0.021 ^a^
SVRI	ephedrine administration	0.011 ^a^
	atropine premedication	0.381
	interaction	0.572

Abbreviations: CI, cardiac index; HR, heart rate; SVI, stroke volume index; SVRI, systemic vascular resistance index. In this model, ephedrine administration, atropine premedication, and their interaction were included as fixed effects, with individual subjects treated as a random effect. ^a^ indicates statistical significance (*p* < 0.05).

## Data Availability

The original contributions presented in this study are included in the article. Further inquiries can be directed to the corresponding author.

## Abbreviations

The following abbreviations are used in this manuscript:

IV	intravenously
SE	saline-ephedrine
AE	atropine-ephedrine
CO	cardiac output
PTA	parasympathetic tone activity
RT	radiation therapy
ECG	electrocardiogram
ASA-PS	American Society of Anesthesiologists Physical Status
HR	heart rate
SpO_2_	percutaneous arterial oxygen saturatio
PETCO_2_	partial pressure of end-tidal carbon dioxide
FIiso	inspiratory isoflurane concentrations
FEiso	expiratory isoflurane concentrations
SAP	systolic arterial pressure
MAP	mean arterial pressure
DAP	diastolic arterial pressure
BL	baseline data
SV	stroke volume
SVI	stroke volume index
CI	cardiac index
SVRI	systemic vascular resistance index
